# Dr. Leonidas H. Laidley

**Published:** 1908-03

**Authors:** 


Dr. Leonidas H. Laidley, of Saint Louis, died at his home February 5, 1908, from cerebral hemorrhage, aged 64 years. Dr. Laidley graduated at Jefferson Medical College, Philadelphia, in 1868, and at Bellevue Hospital Medical College, New York, in 1872. He was professor of gynecology and pelvic surgery in the Marion-Sims-Beaumont Medical College, Saint Louis, a member of the Missouri State Medical Association, of the Saint Louis Medical Society, and medical director of the Louisiana Purchase Exposition in 1904. Dr. Laidley was also a Fellow of the American Association of Obstetricians and Gynecologists.

